# Latent tuberculosis infection is associated with an enrichment of short chain fatty acid producing bacteria in the stool of women living with HIV

**DOI:** 10.21203/rs.3.rs-4182285/v1

**Published:** 2024-04-02

**Authors:** Suventha Moodley, Elouise Kroon, Charissa C. Naidoo, Georgina R. Nyawo, Benjamin G. Wu, Selisha Naidoo, Tinaye L. Chiyaka, Happy Tshivhula, Shivani Singh, Yonghua Li, Robin M. Warren, Eileen G. Hoal, Erwin Schurr, Jose Clemente, Leopoldo N. Segal, Marlo Möller, Grant Theron

**Affiliations:** Stellenbosch University; Stellenbosch University; Stellenbosch University; Stellenbosch University; New York University School of Medicine; Stellenbosch University; Stellenbosch University; Stellenbosch University; New York University School of Medicine; New York University School of Medicine; Stellenbosch University; Stellenbosch University; McGill University; Icahn School of Medicine at Mount Sinai; New York University School of Medicine; Stellenbosch University; Stellenbosch University

**Keywords:** microbiota, latent tuberculosis, HIV, Short chain fatty acids

## Abstract

**Background:**

Latent tuberculosis infection (LTBI) is common in people living with HIV (PLHIV) in high TB burden settings. Active TB is associated with specific stool taxa; however, little is known about the stool microbiota and LTBI, including in PLHIV.

**Method:**

Within a parent study that recruited adult females with HIV from Cape Town, South Africa into predefined age categories (18–25, 35–60 years), we characterised the stool microbiota of those with [interferon-γ release assay (IGRA)- and tuberculin skin test (TST)-positive] or without (IGRA- and TST- negative) LTBI (n=25 per group). 16S rRNA DNA sequences were analysed using QIIME2, Dirichlet Multinomial Mixtures, DESeq2 and PICRUSt2.

**Results:**

No α- or β-diversity differences occurred by LTBI status; however, LTBI-positives were *Faecalibacterium-, Blautia-, Gemmiger-, Bacteroides-enriched and Moryella-, Atopobium-, Corynebacterium-, Streptococcus-*depleted. Inferred metagenome data showed LTBI-negative-enriched pathways included several involved in methylglyoxal degradation, L-arginine, putrescine, 4-aminobutanoate degradation and L-arginine and ornithine degradation. Stool from LTBI-positives demonstrated differential taxa abundance based on a quantitative response to antigen stimulation (*Acidaminococcus-enrichment and Megamonas-, Alistipes-, and Paraprevotella-*depletion associated with higher IGRA or TST responses, respectively). In LTBI-positives, older people had different β-diversities than younger people whereas, in LTBI-negatives, no differences occurred across age groups.

**Conclusion:**

Amongst female PLHIV, those with LTBI had, vs. those without LTBI, *Faecalibacterium, Blautia,* Gemmiger*, Bacteriodes*-enriched, which are producers of short chain fatty acids. Taxonomic differences amongst people with LTBI occurred according to quantitative response to antigen stimulation and age. These data enhance our understanding of the microbiome’s potential role in LTBI.

## Introduction

Tuberculosis (TB) is major cause of death, with 167 000 deaths among persons living with HIV (PLHIV) in 2022^1^. One strategy to prevent active TB is control latent tuberculosis infection (LTBI). LTBI is inferred from a positive tuberculin skin test (TST) or interferon-gamma release assay (IGRA). TB preventive treatment (TPT) strategies play a key role in TB prevention especially in vulnerable populations such as PLHIV.

PLHIV are at greater risk for *Mycobacterium tuberculosis* (MTB) infection and progression to active TB^2^. However, it is poorly understood why some individuals in TB-endemic countries are never infected despite high exposure and why a large proportion of infected individuals never progress. We need more information on correlates of infection and progression, which may have prognostic value.

The microbiome has important immunomodulating effects and the microbiome’s role, including people with LTBI, is an emerging area of interest. For example, in the lung, *Lactobacillus* is enriched in people with LTBI compared to active pulmonary TB group and LTBI-negatives^3^. In the nasopharynx of LTBI-positives, *Staphylococcus* and *Corynebacterium* dominate the microbiome compared to healthy control and active TB cases^4^, and the nasopharyngeal microbiota of LTBI-positives has lower alpha-diversity than LTBI-negatives^5^.

The stool microbiota has potentially an important immunomodulatory role in respiratory disease, including active TB^6^. However, it is comparatively understudied in latent TB. One study among individuals with poorly-controlled diabetes, showed LTBI-positives people to be *Bacteroides*-, *Alistipes*-, and *Blautia*-enriched compared to LTBI-negatives^7^.

During LTBI infection, comparisons of TB cases, HIV-negative LTBI-positive individuals, LTBI-negative and active TB gut microbiomes showed trends of changes in Bacteriodes and Firmicutes, however no significant difference in composition of the stool microbiota^8^. In HIV-negative LTBI-positive individuals showed positive correlation between relative abundances of Coriobacteriaceae and IFN-gamma against MTB antigens more likely associated with of CD4 + T cell^9^. Those studies, however, did not include PLHIV where changes in the gut microbiome are a well-characterised complication of HIV^10^, commonly characterized by reductions in diversity in the stool microbiota^11^. If microbial dysbiosis is detected early in natural history of TB disease (after infection), it may be indicative of early microbial and immune dysregulation associated with subsequent risk of active TB. Tests of progression to active TB are a major public health priority, as is understanding the biological drivers of LTBI. To continue to strengthen our understanding of LTBI and the microbiome, we evaluated the stool microbiota of PLHIV with and without LTBI, nested within a larger study studying resistance to infection.

## Methods

### Recruitment

Participants (18–60 years) were recruited from community health care clinics in Cape Town, South Africa as part of a published parent study (ResisTB)^12^. This cohort was predominantly female, and age was a surrogate for TB exposure, resulting in two groups of 18–25 years and 35–60 years. All people had to be TB symptom screen negative, HIV-positive and stable on ART for ≥ 1 year. Study procedures were approved by the Stellenbosch University Human Research Ethics Committee (N16/03/033A) and each participant provided written informed consent.

### Definitions

IGRA and TST positive (LTBI-positive) people was defined by two QuantiFERON-TB Gold Plus-positive and a positive TST (> 0 mm). IGRA and TST negative (LTBI-negative) was defined by two negative QuantiFERON-TB Gold Plus-negative and a TST (0 mm).

### Microbiota specimen collection and processing

At TST administration, participants were provided with a home stool sampling kit containing an EasySampler (ALPCO, Salem, USA) and a receptacle containing DNA stabilization buffer (Stratec Biomedical, Birkenfeld, Germany). Generally, buffered stools were collected the night before TST reading and returned at TST reading. Upon receipt at the laboratory, buffered stool was frozen at −20°C until batched DNA extraction carried out using the PSP Spin Stool DNA Plus Kit (Stratec Biomedical, Birkenfeld, Germany).

### 16S rRNA gene sequencing and microbiota analysis

V4 region sequencing of the bacterial 16S rRNA gene (150 bp read length, paired-end) was done using Illumina Miseq as described^6^. Sequences was analysed with Quantitative Insights into Microbial Ecology (QIIME2, version 2.0.8). Cluster analysis was carried out using Dirichlet-Multinomial Mixtures (DMM)^13^. Alpha diversity was calculated by Shannon’s diversity with Mann-Whitney testing using GraphPad Prism (v8; GraphPad Software, USA). Beta diversity was calculated using Bray-Curtis with permutational multivariate ANOVA (PERMANOVA) using R (v4.2.2; R Core Team). The functional metagenome was inferred from sequencing data using Phylogenetic Investigation of Communities by Reconstruction of Unobserved States (*PICRUSt2*; v2.0.0)^14^. Differentially abundant taxa and metabolic pathways were identified using *DESeq2* (v1.22.2)^15^ and Benjamini–Hochberg correction adjustment for multiple comparisons (significance level 0.20)^6,16^. For comparisons groups, taxa at higher relative abundance in one group were described as enriched (those at lower relative abundance were described as depleted). For the whole cohort, age and field site correction was applied for *DESeq2* analyses. Linear discriminant analysis (LDA) effect size (LEfSe)^17^ was used to compare the clusters to each other. The proportions test was done using STATA (v18; StataCorp, USA) to determine whether a specific variable was more frequent in different groups. Data and code are available upon publication.

## Results

### Population

**Figure 1:**
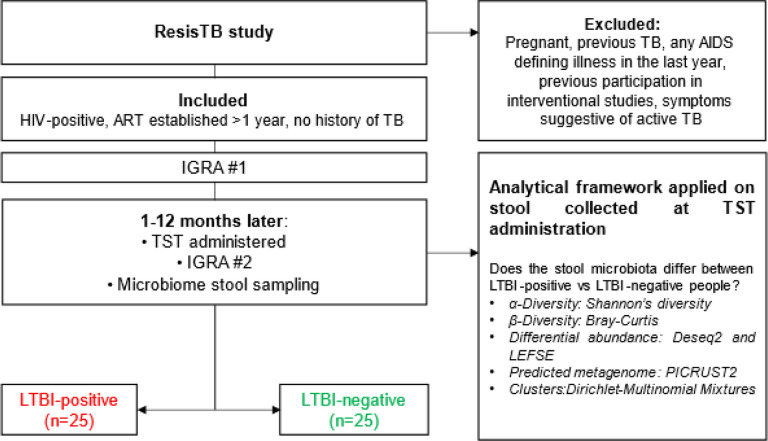
Study profile. We collected and analysed stool from 25 LTBI-positive and 25 LTBI-negative people enrolled in a parent study (ResisTB). HIV: Human immunodeficiency virus; ART: Antiretroviral therapy; IGRA: Interferon gamma release assay; TST: Tuberculin skin test; LTBI: latent TB infection; TB: Tuberculosis.

We collected stool from female PLHIV stable on ART and whom did not have previous TB, with or without LTBI (n = 25 per group; Fig. 1). Demographic data are in Table 1. People with LTBI were younger and more likely to be from Khayelitsha (Site B) Community Health Clinic (Table 1).

Stool from people with LTBI is Moryella-, Atopobium-, Corynebacterium-, Streptococcus-depleted and Faecalibacterium, Blautia, Gemmiger and Bacteriodes-enriched

Overall, no differences were seen by LTBI status for α- (p = 0.168, Fig. 1A) and β-diversity (PERMANOVA, p = 0.841, Fig. 1B). However, Moryella-, Atopobium-, Corynebacterium-, Streptococcus were depleted and Faecalibacterium, Blautia, Gemmiger and Bacteriodes-enriched in LTBI-positives compared to LTBI-negatives (Fig. 1C). People on TB preventative treatment (INH prophylaxis) were, compared to those not on treatment, Blautia-enriched and Moraxella-, Megamonas- and Actinobacillus-depleted (**Supplementary Fig. 1**).

### Comparisons according to age groups (18–25 vs. 35–60 years)

**Figure 2:**
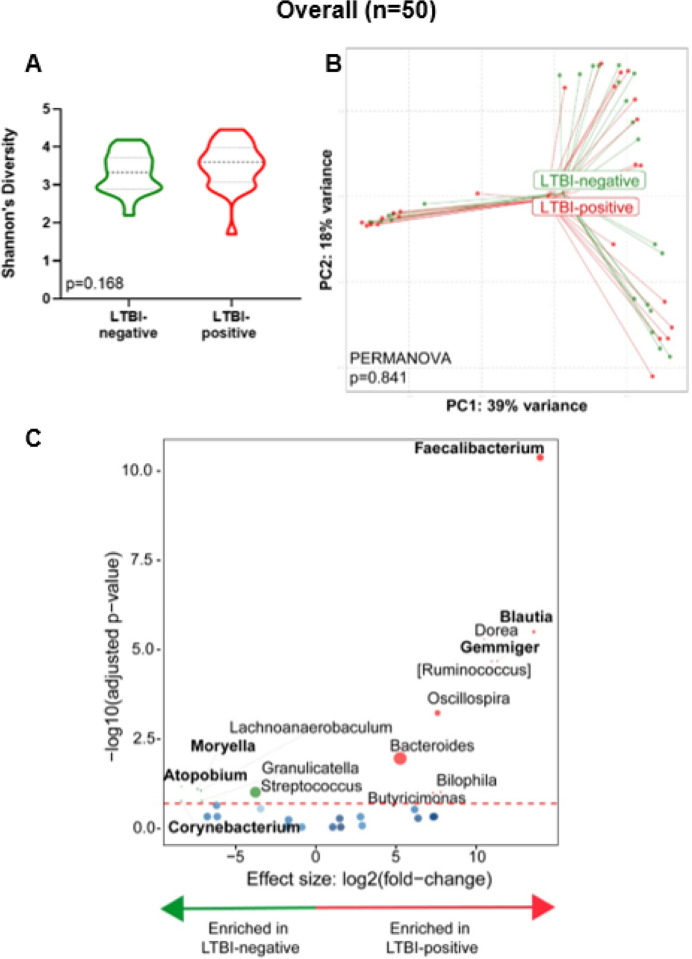
Stool from LTBI-positives is *Moryella*-, *Atopobium*-, *Corynebacterium-Streptococcus*-depleted and *Faecalibacterium, Blautia, Gemmiger* and *Bacteriodes*-enriched. **(A)** Comparison of Shannon’s diversity index of LTBI-positive and LTBI-negative groups. **(B)**Principal coordinate analysis of Bray-Curtis distances between groups. **(C)** Volcano plot depicting differentially abundant taxa. More discriminatory taxa (bolded) appear closer to the left or right, and higher above the threshold (red dotted line, FDR=0.20). Relative abundance of taxa is indicated by circle size. LTBI: Latent TB infection.

**Figure 3:**
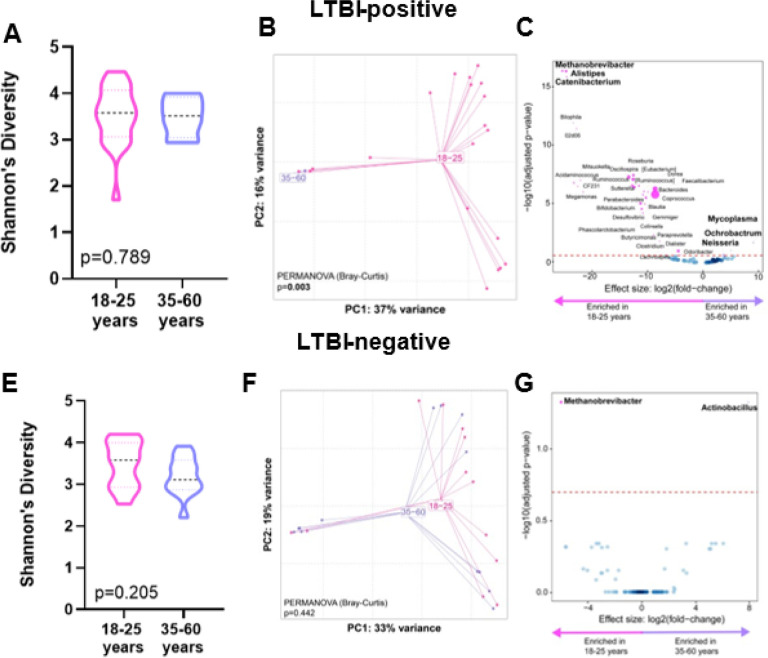
Distinct stool microbiotas by age in LTBI-positive. Shannon’s diversity index, Principal coordinate analysis of Bray-Curtis distances, and volcano plot depicting differentially abundant taxa enriched in 35–60 and 18–25 age groups are for LTBI-positive only (**A, B and C**) and for LTBI-negative only (**E, F and G**). More discriminatory taxa (bolded) appear closer to the left or right, and higher above the threshold (red dotted line, FDR=0.20). LTBI: Latent TB infection.

When we compared age groups within people of the same LTBI status, within LTBI-positive individuals, no α-diversity differences occurred (p = 0.789, Fig. 2A), however, β-diversity differed (p = 0.003, Fig. 2B). Older LTBI-positives were *Ochorobactrum-, Neisseria*- and Mycoplasma-enriched and *Catenibacterium*-, *Alistipes*-, and *Methanobrevibacter*-depleted (Fig. 2C). LTBI-negative individuals did not differ in α-diversity (p = 0.205, Fig. 2E), β-diversity (p = 0.442, Fig. 2F) older LTBI-negatives were also *Methanobrevibacter*-depleted and *Actinobacillus*-enriched. (Fig. 3G). We calculated Bray-Curtis distances for older vs younger LTBI-positives and LTBI-negatives. Both age groups demonstrated similar distances (p = 0.952) across LTBI-statuses (**Supplementary Fig. 2**).

### Distinct metabolic pathway associations with LTBI-positives and -negatives

LTBI-negatives were enriched in methylglyoxal, L-arginine, putrescine, 4-aminobutanoate, and L-ornithine degradation pathways (Fig. 3). LTBI-positives had no differential enrichment.

### Microbial cluster identification and their characteristics

#### Taxonomic analyses

**Figure 5:**
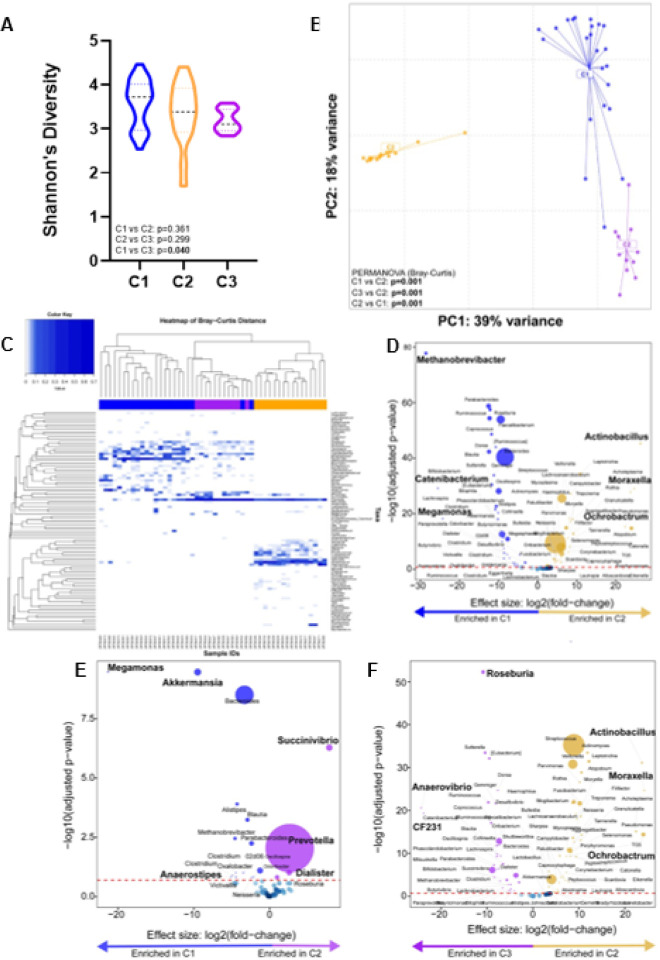
Three distinct microbial clusters, with a high abundance of *Bacteroides, Streptococcus* and *Prevotella*, were identified. Comparison of **(A)** alpha- and **(B)** diversity- by cluster. **(C)** Heatmap shows the composition of each cluster. **(D, E, F)**Volcano plots depicting differentially abundant taxa compared across cluster pairs. More discriminatory taxa (bolded) appear closer to the left or right, and higher above the threshold (red dotted line, FDR=0.20). LTBI: Latent TB infection.

Three clusters (C1, C2 C3) were identified (**Supplementary Fig. 3**) with C1 vs C2 (p = 0.361) and C2 vs C3 (p = 0.299) having similar α-diversity whilst C1 vs C3 differed (p = 0.040) (Fig. 5A). β-diversity differed between clusters (PERMANOVA p = 0.001) (Fig. 5B). C1, C2, and C3 were characterised by high *Bacteriodes*, *Streptococcus*, and *Prevotella* abundances, respectively (Fig. 5C). When cluster pairs were compared (Fig. 5D–F), C1 was, compared to C2, *Methanobrevibacter*-, *Catenibacterium*- and *Megamonas*-enriched and Actinobacillus-, Moraxella- and *Ochrobactrum*-depleted and, compared to C3, *Megamonas-*, *Akkermansia*- and *Anaerostipes*-enriched and *Succinivibrio*-, *Prevotella*-, *Dialister*-depleted. C3 was, compared to C2, *Roseburia*-, *Anaerovibrio*-, and *CF231*-enriched and *Actinobacillus*-, *Moraxella*- and *Ochrobactrum*-depleted. When all three clusters were compared together (**Supplementary Fig. 4**), C1 was, relative to the others, the most enriched in *Bacteroides*, *Oscillospirai* and *Parabacteroides*., C2 most enriched in *Streptococcus*, *Veillonella*, and *Actinomyces*, and C3 most enriched in *Prevotella* and *Catenibacterium*.

#### Clinical and demographic characteristics across clusters

β-diversity differed by field site (**Supplementary table 1**). There were no differences in the proportion of LTBI-positives per cluster (Table 2). C1 and C3 were more likely to be on INH prophylaxis than C2 and more likely to be from sites other than Khayelitsha (Site B) Youth. C2s were, compared to C3s, more likely to be from sites other than Khayelitsha (Site B) CHC Youth and Du Noon CDC.

Within LTBI-positives, taxa are differentially enriched based on the magnitude of the response to antigen stimulation

**Figure 6:**
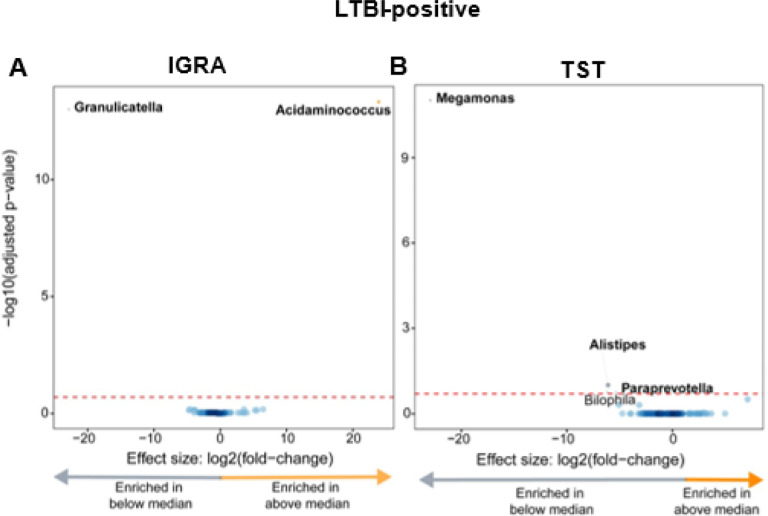
LTBI-positives had, based on the magnitude of the quantitative responses, differentially enriched taxa (those with IGRA responses above the median were *Acidaminococcus*-enriched and *Granulicatella-*depleted, those with TST responses below the median were *Megamonas-*, *Alistipes-, Paraprevotella-*enriched). **(A)** Volcano plot depicting differentially abundant taxa based on the median IGRA quantitative response to antigen (median value 5.56). More discriminatory taxa appear closer to the left or right, and higher above the threshold (red dotted line, FDR=0.20). **(B)**Volcano plot depicting differentially abundant taxa based on the median TST response (Median value 18). LTBI: Latent TB infection.

Taxonomic abundances between quantitative responses to antigen stimulation were compared overall and in LTBI-positives. No differential abundances were identified between the overall cohort above or vs the median average IGRA or TST quantitative response (**Supplementary Fig. 5A and B**). LTBI-positives with an IGRA response above the median were Acidaminococcus-enriched and Granulicatella-depleted (Fig. 6A) and LTBI-positives below the median TST response were *Megamonas*-, *Alistipes*-, and *Paraprevotella*-enriched (Fig. 6B).

## Discussion

We compared, in PLHIV, the stool microbiota of LTBI-positive vs. LTBI-negative women. Our key findings are: 1) stool from LTBI-positives differed from LTBI-negatives in terms of known short chain fatty acid (SCFA)-producing taxa and these were associated with a depletion of metabolite degradation pathways, 2) three taxonomic clusters occurred, characterised by high abundances of *Bacteroides*, *Streptococcus* and *Prevotella*, and clustering associated with INH prophylaxis and health facility location, 3) LTBI-positives with greater quantitative response to mycobacterial antigen stimulation were, vs. LTBI-positives with lesser responses, *Acidaminococcus*-enriched (IGRA readouts) and *Megamonas*-, *Alistipes*-, and *Paraprevotella*-depleted (TST), and 4) β-diversity differed by age group only in LTBI-positives but not - negatives. Our findings help lay a foundation for understanding the microbiome’s role in LTBI.

Stool from people with LTBI was *Moryella*-, *Atopobium*-, *Corynebacterium*-, *Streptococcus*-depleted and *Faecalibacterium, Blautia, Gemmiger* and *Bacteriodes*-enriched. *Faecalibacterium* and *Gemmiger* is a known producer of the butyrate^18^, which is a SCFA that increases incident TB risk^19^. *Bacteroides* produces SCFAs like acetate and propionate^20^. *Blautia* is enriched in people with active TB and independently predicts upregulation of pro-inflammatory pathways^6^. Although the role of *Atopobium* is unclear, Streptococcus, which we found to be depleted in LTBI-positives, produces acetate, which mitigates host inflammation^21^.

Three taxonomic clusters occurred [*Bacteroides* (C1), *Streptococcus* (C2), *Prevotella*-enriched (C3)], however, these were not associated with LTBI status (other studies have documented specific clusters associated with active TB^16^). People within each cluster were more likely to be recruited from different facilities, suggesting potential geographic associations to be considered in future studies. Additionally, C1 was more likely than C2 to be receiving INH prophylaxis. INH prophylaxis itself was associated with *Blautia*-enrichment and *Moraxella*-, *Megamonas*- and *Actinobacillus*-depletion. Other studies have shown *Clostridiales*, *Coprococcus*, *Lachnospiraceae*, and *Ruminococcaceae*-enriched and *Clostridium*_XIVa, *Romboutsia*, and *Roseburia*-depleted stool to occur during rifamycin-based tuberculosis preventive therapy^22^. To our knowledge, our study is the first to show *Blautia*-enriched and *Moraxella*-, *Megamonas*- and *Actinobacillus*-depleted stool in humans on isoniazid TB preventive therapy. This association is interesting as isoniazid itself is a drug thought to have an extremely narrow antimicrobial spectrum (*Mycobacteria* only)^23^.

Additionally, LTBI-positives who had a larger quantitative response to antigen stimulation were, when IGRA readouts were used, *Acidaminococcus*-enriched and *Granulicatella*-depleted, and, when TST readouts was used, *Megamonas*-, *Alistipes*-, *Paraprevotella*-depleted. *Acidaminococcus* produces acetate and butyrate^24^ and is primarily influenced by diet^25^; its enrichment likely reflects lifestyle differences within LTBI-positives. *Paraprevotella* (like *Alistipes*) is a SCFA-producer and generally considered beneficial^26^. This finding is notable because higher quantitative responses are associated with greater risk of incident TB^27^, suggesting such taxa may contribute to this risk, however, this requires prospective confirmation in controlled studies.

Younger people were more likely to be LTBI-positive than older people so, to adjust for age as a potential confounder, we dichotomised people (18–25, 35–60 years). Within LTBI-positives, older vs. younger individuals were enriched in *Ochrobactrum* (an opportunistic pathogen^28^), *Neisseria* (role unclear), *Mycoplasma* (induces a proinflammatory cytokines^29^) and depleted in *Catenibacterium* (enriched in PLHIV^30^ and active TB^31^), *Alistipes* (SCFA producer with potentially anti-inflammatory effects^32^), and *Methanobrevibacter* (a methane producer^33^). Older LTBI-negatives were also *Methanobrevibacter-*depleted and enriched in *Actinobacillus* (inversely associated with amino acid production^34^) but did not show β-diversities differences. This could suggest LTBI results in greater age-related microbiome differences but requires further investigation.

Our study has strengths and limitations. This is a cross-sectional study that, to enhance feasibility, leveraged (but was constrained) the parent ResisTB study. We only evaluated women with HIV and other populations may result in different findings, however, PLHIV do have elevated risk of incident TB. Although people were measured once, our study generates useful data to inform hypothesis-driven interventions to potentially modulate the microbiome.

In conclusion, amongst women living with HIV, those with LTBI were, vs. those without LTBI, primarily differentially abundant in SCFA-producing anaerobic bacteria. Taxonomic differences also occurred amongst people with LTBI by age group, suggesting the age-related microbiome perturbations are more pronounced in LTBI-positives. Longitudinal studies are needed to further delineate the microbiome’s role in LTBI, which this work helps provide a justification for.

## Figures and Tables

**Figure 4:**
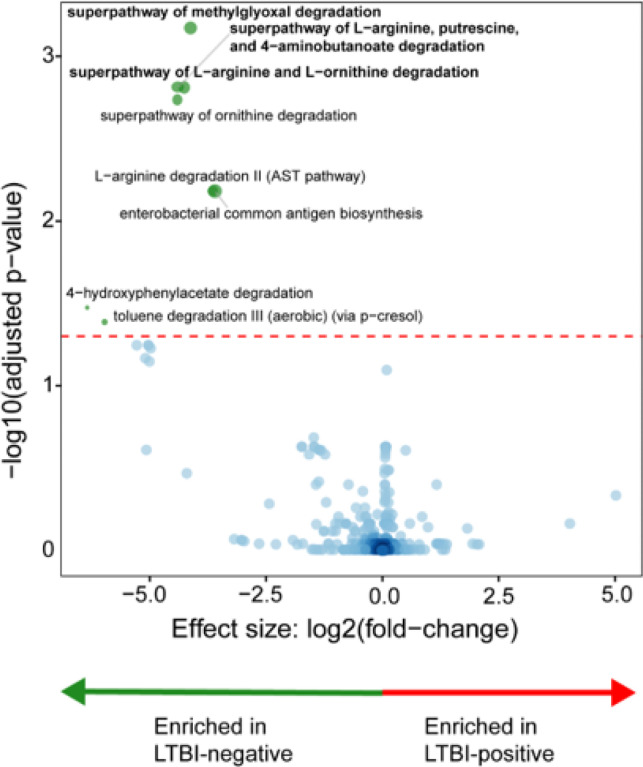
Distinct microbial metabolic pathways are associated with LTBI. LTBI-positives had a depletion of degradation-associated pathways. LTBI: Latent TB infection.

**Table 1:** Demographic and clinical characteristics. People with LTBI were younger and more likely to from Khayelitsha (Site B) CHC. Abbreviations: LTBI: Latent TB infection; BMI: Body mass index; INH: Isoniazid; CHC: Community Health Centre; CDC: Community Day Centre. Data are median (IQR) or n (%).

Characteristic*	Overall (n = 50)	LTBI-positive (n = 25)	LTBI-negative (n = 25)	p-value
Age, years,	24 (23–39)	24 (22–25)	38 (24–41)	**0.005**
18–25	33/50 (66)	21/25 (84)	12/25 (48)	
35–60	17/50 (34)	4/25 (16)	13/25 (52)	**0.007**
CD4 (cells/mm^3^)	468 (368–683)	471 (346–675)	434 (375–705)	0.881
BMI (kg/m)	29 (25–33)	30 (28–34)	26 (24–32)	0.137
Current Tobacco smoker	1/49 (2)	0/24 (0)	1/25 (4)	0.312
Alcohol	31/50 (62)	17/25 (68)	14/25 (56)	0.382
INH prophylaxis	12/50 (24)	6/25 (24)	6/25 (24)	>0.999
Co-trimoxazole prophylaxis	7/50 (14)	2/25 (8)	5/25 (20)	0.221
Field site				
Khayelitsha (Site B) CHC	10/50 (20)	8/25 (32)	2/25 (8)	**0.034**
Khayelitsha (Site B) Youth	7/50 (14)	4/25 (16)	3/25 (12)	0.684
Kraaifontein CHC	11/50 (22)	1/25 (4)	10/25 (40)	**0.002**
Site C Youth	19/50 (38)	12/25 (48)	7/25 (28)	0.145
Du Noon CDC	3/50 (12)	0/25 (0)	3/25 (12)	0.074

* Missing data: Current Tobacco smoker (n = 1)

**Table 2:** Demographic and clinical characteristics of the three clusters found in cohort. C1 was more likely than C2 to be on current INH prophylaxis and more likely to be recruited from Khayelitsha (Site B) CHC, Kraaifontein CHC, Site C Youth or Du Noon CDC. C2 was more likely than C3 to be recruited from Khayelitsha (Site B) CHC, Kraaifontein CHC or Site C Youth. Abbreviations: LTBI: Latent TB infection; BMI: Body mass index; INH: Isoniazid; CHC: Community Health Centre; CDC: Community Day Centre. Data are median (IQR) or n (%).

Characteristics*	C1 (n = 23)	C2 (n = 16)	C3 (n = 11)	p-value C1 vs C2	p-value C2 vs C3	p-value C1 vs C3
Age, years	24 (22–25)	37 (24–40)	25 (24–33)	0.114	0.723	0.146
LTBI-positive	11/23 (48)	9/16 (56)	5/11 (46)	0.601	0.581	0.900
CD4 (cells/mm^3^)	575 (375–700)	395 (372–516)	487 (317–642)	0.242	0.914	0.445
BMI (kg/m)	29 (25–32)	30 (28–35)	27 (23–34)	0.248	0.277	0.612
Current Tobacco smoker	0/23 (0)	0/15(0)	1/11 (9)	-	0.234	0.142
Alcohol	15/23 (65)	9/16 (56)	7/11 (64)	0.574	0.704	0.927
INH prophylaxis	8/23 (35)	1/16 (6)	3/11 (27)	**0.037**	0.130	0.662
Co-trimoxazole prophylaxis	3/23 (13)	4/16 (25)	0/11 (0)	0.337	0.072	0.211
Field site						
Khayelitsha (Site B) CHC	0/23 (0)	10/16 (63)	0/11 (0)	**< 0.001**	**< 0.001**	-
Khayelitsha (Site B) Youth	1/23 (4)	3/16 (19)	3/11 (27)	0.1440	0.597	0.051
Kraaifontein CHC	7/23 (30)	0/16 (0)	4/11 (36)	**0.014**	**0.009**	0.726
Site C Youth	15/23 (65)	0/16 (0)	4/11 (36)	**< 0.001**	**0.009**	0.114
Du Noon CDC	0/23 (0)	3/16 (19)	0/11 (0)	**0.030**	0.127	-

* Missing data: Current Tobacco smoker (n = 1)

## Data Availability

Data is available on reasonable request. Study protocol and datasets generated in this study maybe requested from the corresponding author.

## References

[1] World Health Organization. Global Tuberculosis Report. Geneva, Switzerland., 2023.

[2] Churchyard GJ, Fielding KL, Lewis JJ, A trial of mass isoniazid preventive therapy for tuberculosis control. N Engl J Med. 2014;370(4):301–10. 24450889 10.1056/NEJMoa1214289

[3] Zhang M, Shen L, Zhou X, Chen H. The microbiota of human lung of pulmonary tuberculosis and the alteration caused by anti-tuberculosis drugs. Curr Microbiol. 2022;79(11):321. 36121489 10.1007/s00284-022-03019-9

[4] Huang Y, Tang J-h, Cai Z, Alterations in the nasopharyngeal microbiota associated with active and latent tuberculosis. Tuberculosis. 2022;136:102231. 35964506 10.1016/j.tube.2022.102231

[5] Ruiz-Tagle C, Ugalde JA, Naves R, Araos R, García P, Balcells ME. Reduced microbial diversity of the nasopharyngeal microbiome in household contacts with latent tuberculosis infection. Sci Rep. 2023;13(1):7301. 37147354 10.1038/s41598-023-34052-8 PMC10160714

[6] Naidoo CC, Nyawo GR, Sulaiman I, Anaerobe-enriched gut microbiota predicts pro-inflammatory responses in pulmonary tuberculosis. EBioMedicine. 2021;67:103374. 33975252 10.1016/j.ebiom.2021.103374 PMC8122180

[7] Huang H-L, Luo Y-C, Lu P-L, Gut microbiota composition can reflect immune responses of latent tuberculosis infection in patients with poorly controlled diabetes. Respir Res. 2023;24(1):1–11. 36631857 10.1186/s12931-023-02312-w PMC9835344

[8] Hu Y, Yang Q, Liu B, Gut microbiota associated with pulmonary tuberculosis and dysbiosis caused by anti-tuberculosis drugs. J Infect. 2019;78(4):317–22. 30107196 10.1016/j.jinf.2018.08.006

[9] Huang SF, Yang YY, Chou KT, Fung CP, Wang FD, Su WJ. Systemic proinflammation after Mycobacterium tuberculosis infection was correlated to the gut microbiome in HIV-uninfected humans. Eur J Clin Invest. 2019;49(5):e13068. 30620398 10.1111/eci.13068

[10] Dillon SM, Frank DN, Wilson CC. The gut microbiome and HIV-1 pathogenesis: a two way street. AIDS. 2016;30(18):2737. 27755100 10.1097/QAD.0000000000001289 PMC5101180

[11] Nowak P, Troseid M, Avershina E, Gut microbiota diversity predicts immune status in HIV-1 infection. Aids. 2015;29(18):2409–18. 26355675 10.1097/QAD.0000000000000869

[12] Kroon EE, Kinnear CJ, Orlova M An observational study identifying highly tuberculosis-exposed, HIV-1-positive but persistently TB, tuberculin and IGRA negative persons with M. tuberculosis specific antibodies in Cape Town, South Africa. EBioMedicine 2020; 61: 103053. 33038764 10.1016/j.ebiom.2020.103053 PMC7648124

[13] Holmes I, Harris K, Quince C. Dirichlet multinomial mixtures: generative models for microbial metagenomics. PLoS ONE. 2012;7(2):e30126. 22319561 10.1371/journal.pone.0030126 PMC3272020

[14] Langille MG, Zaneveld J, Caporaso JG, Predictive functional profiling of microbial communities using 16S rRNA marker gene sequences. Nat Biotechnol. 2013;31(9):814–21. 23975157 10.1038/nbt.2676 PMC3819121

[15] Love MI, Huber W, Anders S. Moderated estimation of fold change and dispersion for RNA-seq data with DESeq2. Genome Biol. 2014;15:1–21. 10.1186/s13059-014-0550-8 PMC4302049 25516281

[16] Nyawo GR, Naidoo CC, Wu B, More than Mycobacterium tuberculosis: site-of-disease microbial communities, and their functional and clinical profiles in tuberculous lymphadenitis. Thorax. 2023;78(3):297–308. 36598079 10.1136/thorax-2022-219103 PMC9957952

[17] Segata N, Izard J, Waldron L, Metagenomic biomarker discovery and explanation. Genome Biol. 2011;12(6):1–18. 10.1186/gb-2011-12-6-r60 PMC3218848 21702898

[18] Meng X, Shu Q. Novel primers to identify a wider diversity of butyrate-producing bacteria. World J Microbiol Biotechnol. 2024;40(2):76. 38252387 10.1007/s11274-023-03872-1

[19] Segal LN, Clemente JC, Li Y Anaerobic Bacterial Fermentation Products Increase Tuberculosis Risk in Antiretroviral-Drug-Treated HIV Patients. Cell Host Microbe 2017. 10.1016/j.chom.2017.03.003 PMC5465639 28366509

[20] Fu X, Liu Z, Zhu C, Mou H, Kong Q. Nondigestible carbohydrates, butyrate, and butyrate-producing bacteria. Crit Rev Food Sci Nutr. 2019;59(sup1):S130–52. 30580556 10.1080/10408398.2018.1542587

[21] Vitetta L, Llewellyn H, Old eld D. Gut Dysbiosis and the Intestinal Microbiome: Streptococcus thermophilus a Key Probiotic for Reducing Uremia. Microorganisms 2019; 7(8). 10.3390/microorganisms7080228 PMC6723445 31370220

[22] Séraphin MN, Bellot J, Klann E, Gut microbiota composition and diversity before, during, and two months after rifamycin-based tuberculosis preventive therapy. Sci Rep. 2023;13(1):18933. 37919333 10.1038/s41598-023-44854-5 PMC10622450

[23] Naidoo CC, Nyawo GR, Wu BG, The microbiome and tuberculosis: state of the art, potential applications, and defining the clinical research agenda. Lancet Respiratory Med. 2019;7(10):892–906. 10.1016/S2213-2600(18)30501-0 30910543

[24] Buckel W, Barker H. Two pathways of glutamate fermentation by anaerobic bacteria. J Bacteriol. 1974;117(3):1248–60. 4813895 10.1128/jb.117.3.1248-1260.1974 PMC246608

[25] Zheng J, Hoffman KL, Chen J-S, Dietary inflammatory potential in relation to the gut microbiome: results from a cross-sectional study. Br J Nutr. 2020;124(9):931–42. 32475373 10.1017/S0007114520001853 PMC7554089

[26] Bai J, Li Y, Zhang W, Source of gut microbiota determines oat β-glucan degradation and short chain fatty acid-producing pathway. Food Bioscience. 2021;41:101010.

[27] Hamada Y, Gupta RK, Quartagno M Predictive performance of interferon-gamma release assays and the tuberculin skin test for incident tuberculosis: an individual participant data meta-analysis. EClinicalMedicine 2023; 56. 10.1016/j.eclinm.2022.101815 PMC9829704 36636295

[28] Ryan MP, Pembroke JT. The genus Ochrobactrum as major opportunistic pathogens. Microorganisms. 2020;8(11):1797. 33207839 10.3390/microorganisms8111797 PMC7696743

[29] Chen W, Li D, Paulus B, Wilson I, Chadwick VS. High prevalence of Mycoplasma pneumoniae in intestinal mucosal biopsies from patients with inflammatory bowel disease and controls. Dig Dis Sci. 2001;46:2529–35. 11713965 10.1023/a:1012352626117

[30] Duvallet C, Gibbons SM, Gurry T, Irizarry RA, Alm EJ. Meta-analysis of gut microbiome studies identifies disease-specific and shared responses. Nat Commun. 2017;8(1):1784. 29209090 10.1038/s41467-017-01973-8 PMC5716994

[31] Khaliq A, Ravindran R, Afzal S, Gut microbiome dysbiosis and correlation with blood biomarkers in active-tuberculosis in endemic setting. PLoS ONE. 2021;16(1):e0245534. 33481833 10.1371/journal.pone.0245534 PMC7822526

[32] Oliphant K, Allen-Vercoe E. Macronutrient metabolism by the human gut microbiome: major fermentation by-products and their impact on host health. Microbiome. 2019;7(1):1–15. 31196177 10.1186/s40168-019-0704-8 PMC6567490

[33] Matarazzo F, Ribeiro A, Faveri Md, Taddei C, Martinez MB, Mayer MPA. The domain Archaea in human mucosal surfaces. Clin Microbiol Infect. 2012;18(9):834–40. 22827611 10.1111/j.1469-0691.2012.03958.x

[34] Ling C-w, Miao Z, Xiao M-l, The association of gut microbiota with osteoporosis is mediated by amino acid metabolism: multiomics in a large cohort. J Clin Endocrinol Metabolism. 2021;106(10):e3852–64. 10.1210/clinem/dgab492 34214160

